# Polyploidization as a Retraction Force in Plant Genome Evolution: Sequence Rearrangements in Triticale

**DOI:** 10.1371/journal.pone.0001402

**Published:** 2008-01-02

**Authors:** Miguel Bento, H. Sofia Pereira, Margarida Rocheta, Perry Gustafson, Wanda Viegas, Manuela Silva

**Affiliations:** 1 Centro de Botânica Aplicada à Agricultura, Secção de Genética, Instituto Superior de Agronomia, Technical University of Lisbon, Tapada da Ajuda, Lisboa, Portugal; 2 Curtis Hall, University of Missouri, Columbia, Missouri, United States of America; University of Queensland, Australia

## Abstract

**Background:**

Polyploidization is a major evolutionary process in plants where hybridization and chromosome doubling induce enormous genomic stress and can generate genetic and epigenetic modifications. However, proper evaluation of DNA sequence restructuring events and the precise characterization of sequences involved are still sparse.

**Methodology/Principal Findings:**

Inter Retrotransposons Amplified Polymorphism (IRAP), Retrotransposons Microsatellite Amplified Polymorphism (REMAP) and Inter Simple Sequence Repeat (ISSR) largely confirmed the absence of any intraspecific variation in wheat, rye and triticale. The comparative analysis of banding profiles between wheat and rye inbred lines revealed 34% of monomorphic (common to both parental species) bands for the ten different primer combinations used. The analysis of triticale plants uncovered nearly 51% of rearranged bands in the polyploid, being the majority of these modifications, due to the loss of rye bands (83%). Sequence analysis of rye fragments absent in triticale revealed for instance homology with hydroxyproline-rich glycoproteins (HRGP), a protein that belongs to a major family of inducible defence response proteins. Conversely, a wheat-specific band absent in triticale comprises a nested structure of *copia*-like retrotransposons elements, namely *Claudia* and *Barbara*. Sequencing of a polyploid-specific band (absent in both parents) revealed a microsatellite related sequence. Cytological studies using Fluorescent *In Situ* Hybridization (FISH) with REMAP products revealed a widespread distribution of retrotransposon and/or microsatellite flanking sequences on rye chromosomes, with a preferential accumulation in heterochromatic sub-telomeric domains.

**Conclusions/Significance:**

Here, we used PCR-based molecular marker techniques involving retrotransposons and microsatellites to uncover polyploidization induced genetic restructuring in triticale. Sequence analysis of rearranged genomic fragments either from rye or wheat origin showed these to be retrotransposon-related as well as coding sequences. Further FISH analysis revealed possible chromosome hotspots for sequence rearrangements. The role of chromatin condensation on the origin of genomic rearrangements mediated by polyploidization in triticale is also discussed.

## Introduction

Polyploidization is an evolutionary process where two or more genomes are joined into the same nucleus. There are numerous examples of polyploidization in the plant kingdom, where chromosome fusions/fissions and rearrangements occurred following speciation. In fact, many modern day diploids are believed to have arisen from ancient polyploidization events (reviewed in [1]). Polyploidization is responsible for the emergence of genotypic plasticity, providing polyploids with the ability to tolerate genomic variations better than their diploid progenitors. This dynamic process has been explored in cereal species, which share a monophyletic origin. Although phylogenetic and molecular studies demonstrated large differences in genome size between cereal species, gene number and synteny are conserved (reviewed in[2]). Comparisons at the genetic map level show high genome colinearity conservation between cereal species, occasionally disrupted by gross chromosomal translocations [3].

Polyploid formation places the hybrid under a considerable amount of stress and/or genetic shock, which in turn can leads to a number of genetic and epigenetic modifications. Solely genetic changes include translocations and transpositions as well as sequence deletions and insertions, while epigenetic changes take into account non-additive gene regulation, transposon transcription, silencing or sub-funtionalization of homoeologous genes, and chromatin condensation [4]–[6]. Previous studies on wild wheat polyploid relatives, such as *Triticum* and *Aegilops* spp., suggested that the genetic and epigenetic changes that occurred were not random, but rather directed and reproducible [7]–[11]. Directed and stable modifications have also been reported in *Brassica* [12] as well as in *Arabidopsis* [13]. In the allopolyploid *A. suecica,* a product of hybridization of *A. thaliana* and *A. arenosa*, genetic changes involving the loss of one parental-specific rDNA *locus* were observed in both naturally occurring as well as synthetic polyploids [14], [15]. Genome rearrangements have also been extensively studied in triticale, a man-made wheat (*Triticum* ssp.)/rye (*Secale cereale* L.) allopolyploid (X *Triticosecale* Wittmack) [16], [17]. Amplified fragment length polymorphism (AFLP) and restriction fragment length polymorphism (RFLP) analyses have established the occurrence of genetic and epigenetic modifications in triticale, mostly attributable to the rye parental genome [17]. Moreover, most of the modifications revealed by AFLP occurred to a greater degree immediately after hybridization, especially concerning sequences from rye origin, compared to the continuous variation events that occurred at a very small rate following chromosome duplication [18]. Using the same techniques on newly formed polyploids of *Aegilops* and *Triticum*, rapid alterations of chromosome- and genome-specific sequences were demonstrated, with the preferential loss of parental bands [8], [9]. It must be emphasized here that both AFLP and RFLP analyses were performed utilizing non-methylation and methylation sensitive restriction enzymes, thereby uncovering differences which cannot be attributable solely to sequence modifications.

**Figure 1:**
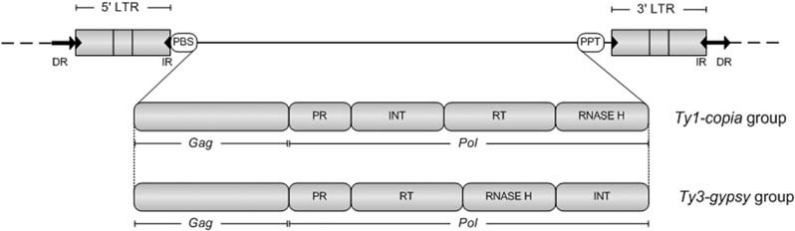
Structural features of *copia* and *gypsy* retrotransposons. LTR, long terminal repeat; Gag, core structural protein genes; PR, protease; RT, reverse transcriptase; INT, integrase; PBS, primer binding site; PPT, polypurine tract; DR, direct repeat; IR, inverted repeat (adapted from [20]).

The exact level of DNA sequence restructuring events involved in this important evolutionary process remains to be determined. In addition, precise information regarding the actual sequences involved is scarce. In order to better understand the genomic processes underlying polyploidization, microsatellite and retrotransposon PCR-based molecular marker techniques were utilized to evaluate exclusively genetic rearrangements in triticale. Microsatellites, or Simple Sequence Repeats (SSRs), are polymorphic *loci* present in nuclear DNA that consist of repeating units of 1–6 base pairs in length. They are typically neutral, co-dominant and widely spread throughout the genome. Retrotransposons are ubiquitous in the plant kingdom, being the main constituent of large plant genomes [19]. These transposable genetic elements require the action of reverse transcriptase on an RNA intermediate to integrate in the host genome by a ´copy and pasté method of transposition [20]. They are conventionally divided into groups, depending whether or not they possess long terminal repeats (LTRs). The LTR retrotransposons are further classified into the *Ty1- copia* and *Ty3-gypsy* families (Figure 1). Due to their dynamics and mobility, it is widely accepted that these elements generate molecular modifications and increase genome size, and therefore have an important role in genome evolution and speciation. Actually, an increase in Retrotransposon-related transcripts has been detected in both wheat and Arabidopsis synthetic polyploids [7], [13], [21], [22], although actual transposition of these elements has never been proved in newly synthesized polyploids.

In this study, we demonstrate that Inter Retrotransposons Amplified Polymorphism (IRAP), Retrotransposons Microsatellite Amplified Polymorphism (REMAP), and Inter Simple Sequence Repeat (ISSR) are powerful tools for polyploid analysis. A high rate of polyploidization induced DNA sequence rearrangements in retrotransposon and/or microsatellite associated sequences were uncovered in triticale. These techniques also permitted the isolation of restructured fragments for sequence analysis, unambiguously demonstrating that repetitive and coding sequences are involved in the evolutionary process mediated by polyploidization. FISH for chromosome mapping of sequences associated to the retrotransposon Nikita and/or to the microsatellite (CT)9G showed these to be widely distributed throughout all rye chromosomes, with preferential localization in major heterochromatic domains, suggesting that chromatin condensation plays an important role in polyploid evolution.

## Results

### IRAP, REMAP and ISSR profiles enhance wheat and rye phylogenetic relationships

**Figure 2:**
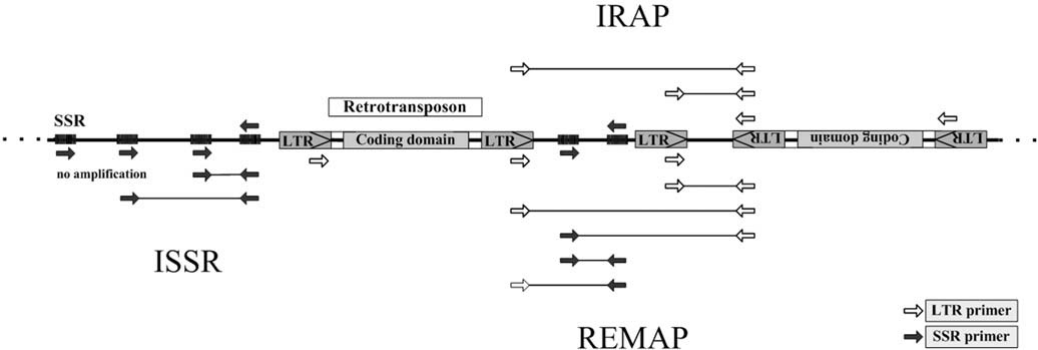
Principles of the IRAP, REMAP and ISSR procedures. IRAP: LTR primers (white arrows) facing outward from the ends of LTRs will amplify intervening DNA from retrotransposons or loose LTRs in opposite orientations. Retrotransposons or loose LTRs in the same orientation will not result in amplification products. REMAP: LTR primers are used together with SSR primers (black arrows), the products of amplification will be the combination of all retrotransposons or loose LTRs and SSRs in opposite orientations, sites with the same orientation will not result in amplification products. ISSR: SSR primers (black arrows) will amplify intervening DNA from SSRs in opposite orientations; sites with the same orientation will not result in amplification products.

To test if the IRAP, REMAP and ISSR techniques constitute efficient tools to evaluate genomic rearrangements within allopolyploid triticale, we initially analyzed the banding profiles obtained with different primer combinations in wheat and rye parental plants. Seven primer combinations were tested with IRAP and REMAP using the primer for the LTR sequence of the barley retrotransposon Nikita by itself as well as combined with three LTR primers from additional barley retrotransposons (Sabrina, Sukkula, and Stowaway), and with three anchored microsatellite primers (GA)9C, (CT)9G, and (CA)9G (Table 1). ISSR banding profiles were obtained for all three di-nucleotide repeats. In order to reduce the potential number of fragments amplified and obtain consistent, easily analyzable and reproducible banding patterns, a single LTR primer from each retrotransposon was utilized on all IRAP and REMAP combinations (Figure 2). The banding profiles yielded a considerable number of distinct and reproducible bands in all the lines and for all primer combinations analyzed. Only bands between 100 and 1650 bp were scored, as this gel region produced the highest quality profiles, allowing for discrimination of major bands against a low background. Qualitative differences between profiles and minor non-reproducible IRAP, REMAP and ISSR bands were not considered. Differences in intensities between bands were obvious within the same species for each primer combination but did not show any direct relation with the size of the amplified fragment.

**Table 1:** Primers used for PCR analysis.

Primer		Sequence
**LTR**	Barley Retrotransposon	
C0699	Nikita	5′-CGCTCCAGCGGTACTGCC
C0945	Sabrina	5′-GCAAGCTTCCGTTTCCGC
9900	Sukkula	5′-GATAGGGTCGCATCTTGGGCGTGAC
Stowaway	Stowaway	5′-CTTATATTTAGGAACGGAGGGAGT
**SSR**		
(GA)9C		5′-GAGAGAGAGAGAGAGAGAC
(CT)9G		5′-CTCTCTCTCTCTCTCTCTG
(CA)9G		5′-CACACACACACACACACAG

**Figure 3:**
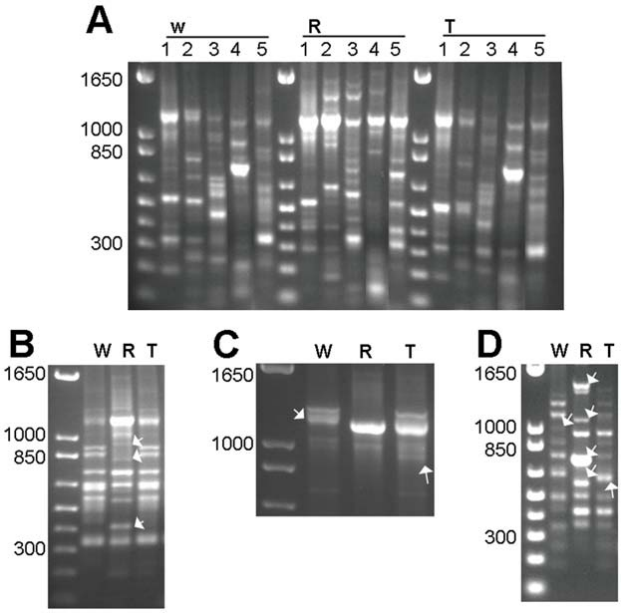
IRAP, REMAP and ISSR banding profiles of wheat (W), rye (R), triticale (T). (a) grouped by species, obtained with IRAP and REMAP utilizing primer Nikita (1), Nikita/Sabrina (2), Nikita/Sukkula (3), Nikita/(GA)9C (4), Nikita/(CA)9G (5). (b) Banding profiles obtained with REMAP utilizing primer combination Nikita/(CA)9G, arrows indicate the three polyploid rearranged bands of rye genome origin absent in triticale. (c) Detailed section of banding profiles obtained with IRAP utilizing primer Nikita, arrows indicate a rearranged band of wheat genome origin absent in triticale and a novel band in triticale. (d) Banding profiles obtained with ISSR utilizing primer combination (CT)9G/(CT)9G, arrows indicate five rearranged bands in the polyploid, one of wheat genome origin absent in triticale, four of rye genome origin absent in triticale and one novel band in triticale.

Banding profiles from wheat and rye genomes obtained with five of the ten primer combinations are shown in Figure 3a. The IRAP and REMAP results are summarized in Table 2 and the ISSR results in Table 3. The total number of bands per species as well as the number of monomorphic (similar in both parental species) and polymorphic (observed in only one parental species) bands observed for all ten primer combinations are shown. Although our results confirm the phylogenetic proximity between wheat and rye genomes, the number of polymorphic bands obtained with IRAP and REMAP was nevertheless 65% (74 of 114 total number of bands observed in wheat and rye) and 68% (39 of 57 total number of bands observed in wheat and rye) with ISSR, proving these markers are excellent tools to discriminate between closely related species. Intraspecific comparisons were also performed through the analysis of three distinct wheat and rye plants using primer Nikita (Supporting information Figure S1). The banding profiles were identical for all plants of the same species, confirming their high inbreed nature.

**Table 2:** IRAP and REMAP analysis in triticale allopolyploid and its diploid progenitors.

Primer combined with Nikita	Number of bands with different primer combinations							
	IRAP				REMAP			
	Nikita	Sabrina	Sukkula	Stowaway	(GA)9C	(CT)9G	(CA)9G	Total
Wheat	7	7	11	4	6	9	12	56
Rye	5	9	13	8	5	8	10	58
** Monomorphic** [i]	1	1	7	2	2	-	7	** 40** (35%)
** Polymorphic** [ii]	10	14	10	8	7	17	8	**74** (65%)
Polyploid								
**Expected** [iii]	11	15	17	10	9	17	15	**94**
**Conserved** [iv]	9	10	14	6	7	11	12	**69** (73%)
**Observed**	10	10	14	6	8	13	12	**73** [ii]
Polyploid **rearranged** [v]	3	5	3	4	3	8	3	**29**
**Eliminated** from wheat	1	1	1	1	-	1	-	**5** (17%)
**Eliminated** from rye	1	4	2	3	2	5	3	**20** (69%)
** Novel** in triticale	1	-	-	-	1	2	-	**4** (14%)

[i] Monomorphic bands: common to both triticale progenitors [40 (20 wheat+20 rye)/114 (56 wheat+58 rye) = 35.1%];

[ii] Polymorphic bands: observed in only one triticale progenitor;

[iii] Polyploid expected bands: the ones expected as an addition pattern of parental bands observed;

[iv] Polyploid conserved bands: parental bands observed in the polyploid;

[v] Polyploid rearranged bands: parental bands absent in the polyploid and novel bands only observed in the polyploid.

**Table 3:** ISSR analysis in triticale allopolyploid and its diploid progenitors.

	Number of bands with different di-nucleotide primers			
	ISSR			
	(GA)9C	(CT)9G	(CA)9G	Total
Wheat	13	12	5	30
Rye	14	9	4	27
** Monomorphic** [i]	7	2	-	**18** (32%)
** Polymorphic** [ii]	13	17	9	**39** (68%)
Polyploid				
** Expected** [iii]	20	19	9	**48**
C**onserved** [iv]	14	14	9	**37** (77%)
O**bserved**	15	15	9	**39**
Polyploid **rearranged** [v]	7	6	-	**13**
** Eliminated** from wheat	-	1	-	** 1** (8%)
** Eliminated** from rye	6	4	-	**10** (77%)
** Novel** in triticale	1	1	-	**2** (15%)

[i] Monomorphic bands: common to both triticale progenitors [18 (9 wheat+9 rye)/57 (30 wheat+27 rye) = 32%];

[ii] Polymorphic bands: observed in only one triticale progenitor;

[iii] Polyploid expected bands: the ones expected as an addition pattern of parental bands observed;

[iv] Polyploid conserved bands: parental bands observed in the polyploid;

[v] Polyploid rearranged bands: parental bands absent in the polyploid and novel bands only observed in the polyploid.

Comparison of banding profiles between primer combinations revealed that they were reproducible and unique to each primer combination. However, profiles resulting from combinations of primers did not necessarily result in additive profiles of reactions in which each primer is used alone. For instance, the entire banding pattern obtained with primer Nikita was not observed in the other IRAP and REMAP reactions where this primer was utilized in combination with other microsatellite or retrotransposon primer. Since the results obtained from three technical replicates for each PCR experiment were identical the amplification products obtained in each reaction seem therefore to represent a reproducible competition between the primer and its binding sites for each specific reaction mixture. Analyzing banding profiles of all the reactions tested resulted in the following maximum and minimum number of bands: (i) IRAP and REMAP bands observed in wheat and rye ranged between a minimum of four in wheat (primer combination Nikita/Stowaway) and a maximum of thirteen in rye (primer combination Nikita/Sukkula); (ii) the number of monomorphic bands ranged between zero (primer combination Nikita/(CT)9G) and seven (primer combinations Nikita/Sukkula and Nikita/(CA)9G); (iii) with ISSR, the number of bands observed in wheat and rye ranged between a minimum of four in rye (with primer (CA)9G) and a maximum of fourteen in rye (with primer (GA)9C), with the number of monomorphic bands ranging between zero (with primer (CA)9G) and seven (with primer (GA)9C).

### Parental genomic rearrangements in triticale

Since the parental materials were strictly inbred over many years, any genomic changes revealed in the triticale should be directly related to genome responses to polyploidization. In fact, genomic rearrangements were observed as non-additive IRAP, REMAP and ISSR banding profiles in triticale compared to those of the wheat and rye parental species. All seven IRAP and REMAP and two of the three ISSR primer combinations showed the occurrence of rearrangements in triticale (Figure 3a, Table 2 and 3) which resulted from the loss of parental bands in triticale or, conversely, novel bands appearing in the allopolyploid that were not seen in the parental genomes. Moreover, the presence in the banding profile obtained with primer Nikita for the parental genome mixture (Supporting information Figure S2) of the triticale rearranged bands (lost parental bands), confirmed that the genome modifications revealed resulted from polyplidization. On the other hand, to evaluate if the restructuring events detected in the polyploid genotype were present in all plants analyzed, IRAP utilizing primer Nikita was performed with DNA extracted from three triticale plants (Supporting information Figure S1) .The individual banding profiles clearly demonstrated no differences between individuals, indicating that triticale plants are stable for the molecular markers selected.

To evaluate the frequency of genome rearrangements in triticale, the number of polymorphic wheat and rye bands was added to the number of monomorphic bands (a band present in both parental profiles was counted only once), and the result was compared with the number of bands observed in triticale profiles. These calculations demonstrated that only 73% (69 bands observed/94 expected) of the parental bands were conserved in triticale with IRAP and REMAP (Table 2), and 77% (37 bands observed /48 expected) with ISSR (Table 3). Out of the seven IRAP and REMAP primer combinations analyzed, a total of twenty-nine rearrangements were detected in triticale, representing the loss of five wheat origin bands (17%) and twenty rye origin bands (69%), as well as the appearance of four novel bands (14%) not seen in either parental species. The rearranged bands observed with the seven IRAP and REMAP experiments were as follows: (i) IRAP with primer Nikita resulted in the loss of one band from rye, the loss of one band from wheat and the appearance of one novel band (Figure 3c); (ii) primer combination Nikita/Sabrina resulted in the loss of four bands from rye and the loss of one band from wheat; (iii) with IRAP primer combination Nikita/Sukkula the loss of two bands from rye and the loss of one band from wheat was observed; (iv) IRAP utilizing primer combination Nikita/Stowaway resulted in the loss of three bands from rye and the loss of one band from wheat; (v) REMAP utilizing primer combination Nikita/(GA)9C resulted in the loss of two bands from rye and the appearance of one novel band; (vi) primer combination Nikita/(CT)9G revealed the loss of five bands from rye, the loss of one band from wheat and the appearance of two novel bands in triticale; and (vii) primer combination Nikita/(CA)9G indicated the loss of three rye origin bands in triticale (Figure 3b). Thirteen rearranged bands were identified in triticale with the three ISSR primers tested, corresponding to loss of one wheat origin band (8%) and from the loss of ten rye origin bands (77%) and the occurrence of two novel bands (15%) not present in either of the parental species (Table 3). The rearranged bands observed with the three ISSR experiments were as follows: (i) ISSR with primer (GA)9C resulted in the loss of six bands from rye and the appearance of one novel band; and (ii) primer (CT)9G indicated the loss of four bands from rye, one band from wheat and the appearance of one novel band in the triticale (Figure 3d).

Analyzing the total number of IRAP, REMAP and ISSR rearrangements clearly indicated that the loss of parental bands in triticale occurred much more frequently (43%) than the appearance of novel bands (7%), (Table 4). Amongst triticale lost bands, most involved rye specific bands (10 from IRAP, 10 from REMAP and 10 from ISSR reactions), comparing to the loss of six wheat specific bands (4 from IRAP; 1 from REMAP and 1 from ISSR reactions). The loss of a monomorphic band (common to wheat and rye progenitors) was never observed in triticale. Conversely, a total of six novel bands were detected in the allopolyploid with the molecular markers used (1 with IRAP, 3 with REMAP and 2 with ISSR, respectively).

**Table 4:** Summary of rearrangements identified in triticale by IRAP, REMAP, and ISSR.

	Polymorphic bands observed	Rearranged bands (% of observed)		
		Eliminated	Novel	TOTAL
**IRAP**	29	14 (48%)	1 (3%)	**15 (52%)**
**REMAP**	24	11 (46%)	3 (13%)	**14 (58%)**
**ISSR**	30	11 (37%)	2 (7%)	**13 (43%)**
**Total**	83	36 (43%)	6 (7%)	**42 (51%)**

### Sequences analysis of polyploid rearranged bands

Three rearranged fragments uncovered in triticale arising from IRAP and REMAP with three different primer combinations were gel-isolated, purified and cloned for sequence analysis. These included one band from the wheat parental genome absent in triticale, one band from the rye parental genome missing in the polyploidy, as well as a novel triticale-specific band. Sequence analyses of these three rearranged bands is described below.

#### A putative copia-like retrotransposon

**Figure 4:**
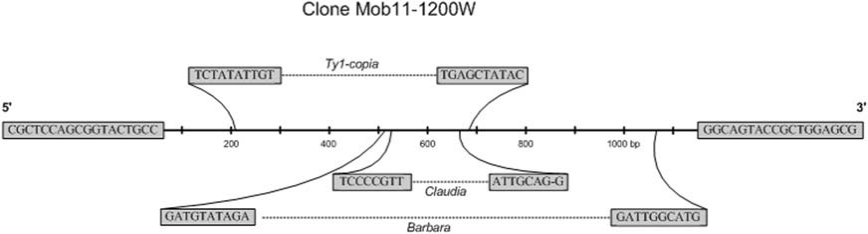
Structure of putative partial retrotransposon-like sequence Mob-11-1200 from *T. aestivum* with 1200 kb. The terminal boxes at each end represent the primer sequence used in the amplification (C0699). Shown in the expansions are three *copia*-like retroelements that seem to have partial inserts inside clone Mob11-1200. Boxes indicate the beginnings and the ends of these retrotransposons. Accession numbers: *Barbara* retrotransposon (*T. monococcum*, AF326781); *Claudia* retrotransposon (gi:18496650); *Ty1-copia* retrotransposon (*O. sativa* gi:.57114405).

**Figure 5:**
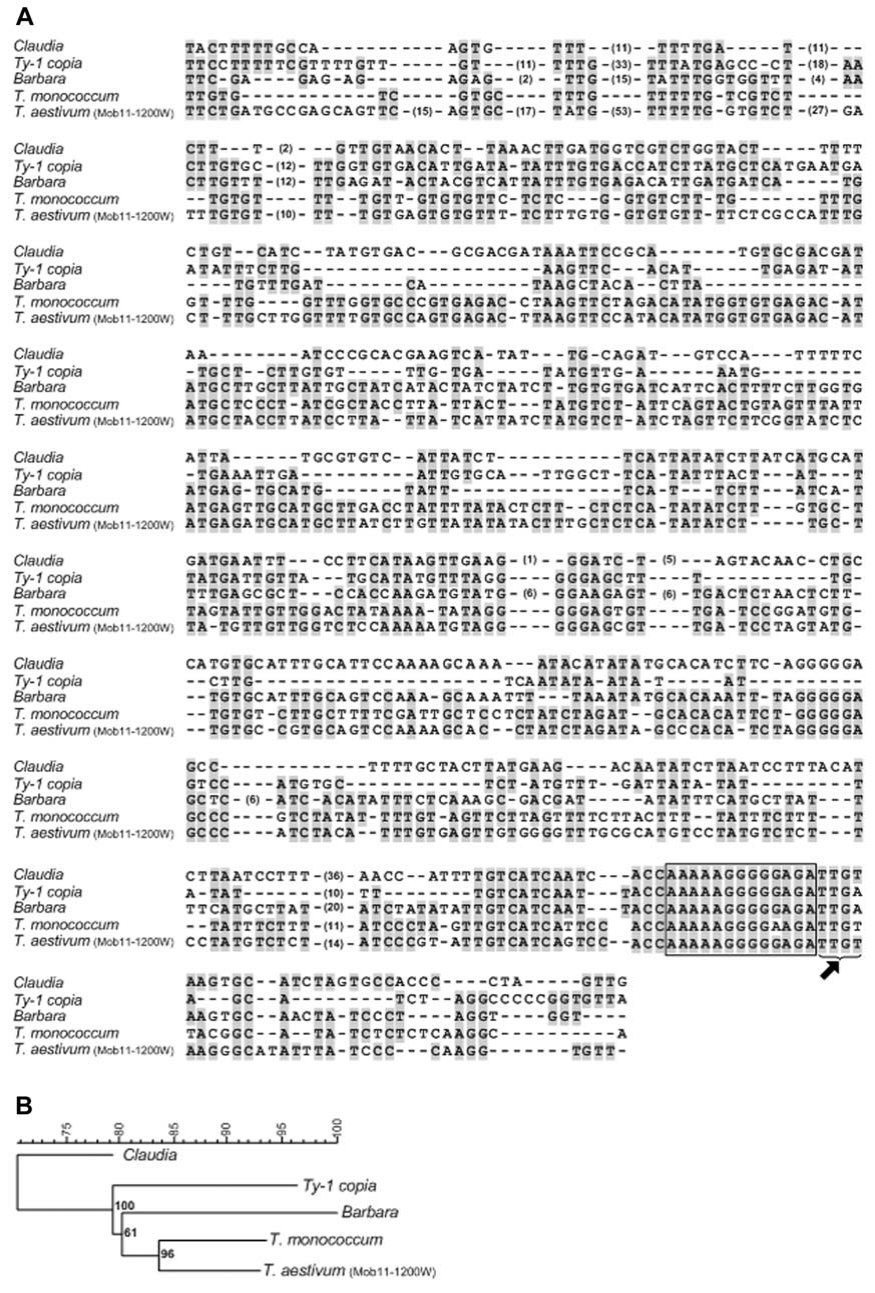
Sequence alignment of the clone Mob-11-1200 from *T. aestivum* with several *copia* retrotransposons. (a) Only partial sequences, corresponding to the conserved parts of *Pol* region are shown. The polypurine tract (PPT) are boxed and arrow indicate the inverted repeat (IR) signature just before the beginning of the 3′ LTR. Numbers in brackets indicate the number of nucleotides separating the sequences. (b) Based on previous nucleotide alignment, a tree was constructed utilizing the Neighour-Joining method [24]. The numbers on the branches represent bootstrap support for 1,000 replicates. Numbers in bold represent cophenetic correlations, which confirm the stability of the tree nodes. Sequences were aligned using Bionumerics software (version 3.5). Accession numbers: *Barbara* retrotransposon (*T. monococcum*, AF326781); *Claudia* retrotransposon (gi:18496650); *Ty1-copia* retrotransposon (*O. sativa* gi: 57114405); *T. monococcum* 7Am (AF488415).

IRAP using only the Nikita primer resulted in a 1219 bp rearranged fragment of wheat origin absent in triticale, which was named MoB-11-1200W (accession number EF486520). Utilizing NCBI, TIGR, and PlantSat (repetitive plant sequences) databases, nucleotide alignments show 71, 79, 80 and 84% homology with *copia*-like retrotransposons *Claudia*, *Ty1* from *O. sativa*, *Barbara* and an unnamed retrotransposon from *T. monococcum* 7Am, respectively (Figure 4). Immediately before the 3′ LTR, MoB-11-1200W presented a short polypurine tract (PPT) with 13 purines (AAAAAGGGGGAGA) (Figure 5a) and downstream of the PPT an inverted repeat with four nucleotides (TTGT) indicative of the beginning of 3′ LTR. Neither in the clone or in the aligned retroelements was found between the polypurine tract and the right LTR, a dinucleotide characteristic of the LTR end-sequence of retroviruses and retrotransposons [23]. This absence seems to be a characteristic of this retroelement group. Based on the nucleotide alignment, a phylogenetic consensus tree (Figure 5b) was constructed using the Neighbor-Joining method [24] on the basis of a distance matrix calculated with the Bionumerics software (version 3.5). There was one major clade supported by a bootstrap value of 100% that included all the elements except *Claudia*, which appears as an out-group. Inside the major clade two clades supported by bootstrap values of 61 and 96% were selected, the first one including the retroelement *Barbara* from *T. monococcum* and the second with *T. monococcum 7 Am* and Mob-11-1200W clone.

#### An hydroxiproline-rich glycoprotein sequence

**Figure 6:**
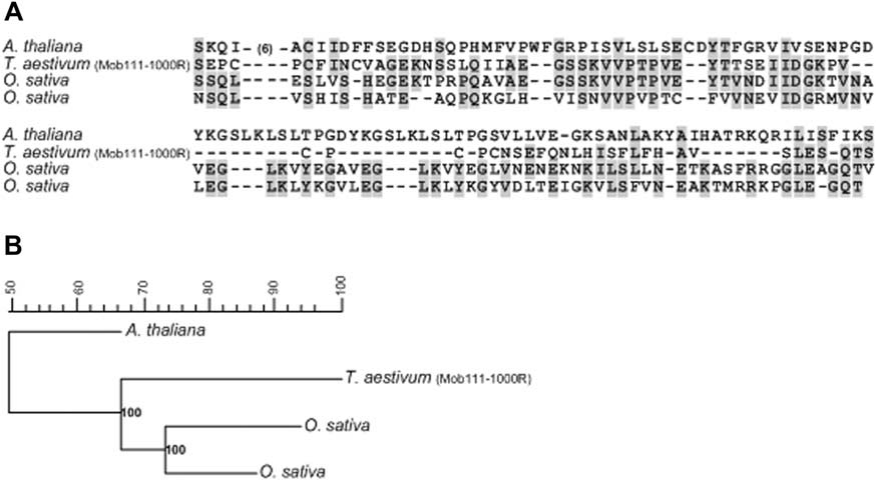
Alignments of conceptual translation of clone Mob-111-1000R. (a) The alignment shows homology with sequences coding for a hydroxiproline-rich glycoprotein. (b) Based on the alignment of amino acid residues, the tree was constructed utilizing the Neighour-Joining method [24]. The numbers on the branches represent bootstrap support for 1,000 replicates. Sequences were aligned using Bionumerics software (version 3.5). Accession numbers: *A. thaliana* (gi: 18394153); *O. sativa* (gi:20270098); *O. sativa* (gi: 110288545).

The 963 bp rearranged band of rye parental origin absent in triticale was isolated from the REMAP Nikita/(CA)9G) and named MoB-111-1000R (accession number EF486521). The aminoacid residues were tested against the NCBI, TIGR, and PlantSat (repetitive plant sequences) databases (Figure 6a). The alignment shows 50 and 73% homology with several sequences that code for a hydroxyproline-rich glycoprotein from *A. thaliana* and *O. sativa*, respectively. Based on the aminoacid alignment (Figure 6b), a phylogenetic consensus tree was constructed using the Neighbor-Joining method [24] on the basis of a distance matrix calculated with the Bionumerics software (version 3.5). The tree showed *A. thaliana* as an out-group and one major clade supported by a bootstrap value of 100% that included clone Mob-111-1000R and two independent sequences from *O. sativa*.

#### A characteristic triticale microsatellite

REMAP with primer combination Nikita/(CT)9G resulted in a novel 100 bp triticale specific fragment that was not seen in either of the parental genomes, named Mob-110-100T which revealed to be mainly composed of microsatellites, and did not produce any significant alignment against the databases.

### Chromosome mapping of Nikita LTR and microsatellite flanking sequences in rye

**Figure 7:**
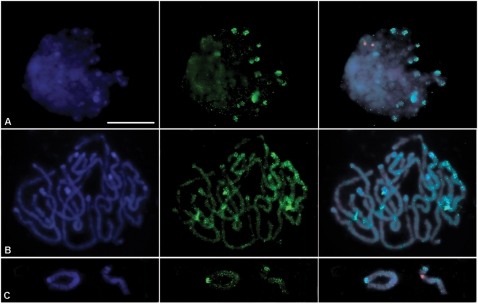
FISH on rye meristematic root-tip cells with REMAP product and pTa71. The following probes were used: total amplification product of REMAP reaction with primer combination Nikita/(CT)9G (green signal) and rDNA unit pTa71 (red signal). DNA was counterstained with DAPI (blue). (a) Interphase); (b) Prometaphase; and (c) Metaphase chromosomes. Bar 10 µm.

In order to characterize the genomic distribution of the amplified products, the complete REMAP amplification reaction with primer combination Nikita/(CT)9G as well as the rDNA unit from wheat (pTa71) were utilized as probes to perform fluorescent *in situ* hybridization (FISH) on rye. The REMAP probe was labelled with digoxigenin and detected with anti-digoxigenin FITC conjugated (green), the rDNA probe was labelled with biotin and detected with streptavidin Cy3 conjugated (red) and DAPI was used as a DNA counterstain (Figure 7). Spreads of meristematic root-tip cells allowed for the visualization of specific REMAP sequences throughout the cell cycle, namely on interphase nuclei (Figure 7a), prometaphase (Figure 7b) and metaphase chromosomes (Figure 7c). In rye interphase nuclei, the nuclear Rabl organization of chromosomes was evident as low intensity DAPI staining in one nuclear pole clearly contrasted to large DAPI-positive signals characteristic of heterochromatic sub-telomeric domains observed in the opposite pole (Figure 7a). Detailed analysis of interphase nuclei shows ten heterochromatic DAPI positive domains co-localizing with the most intense aggregation of FISH REMAP signals and numerous dot-like signals dispersed throughout the entire nucleus. Due to chromosome condensation a widespread distribution of FISH signals was observed throughout rye prometaphase chromosomes (Figure 7b). It was possible to distinguish two signals on both chromatids in some interstitial domains, as well as a marked sub-telomeric accumulation on most chromosomes. Rye metaphase chromosomes clearly demonstrated absence of signal in centromeric and rDNA domains and on sub-telomeric regions that are not DAPI positive (Figure 7c). Taken together, FISH analysis of sequences amplified by REMAP with Nikita/(CT)9G showed a wide distribution throughout the rye genome although preferentially accumulated at heterochromatic domains.

## Discussion

Retrotransposon rich genomic domains are volatile and susceptible to a variety of responses to diverse stresses, such as those induced by polyploidization. In this study, large scale screening of retrotransposon and microsatellite rich genomic regions using IRAP, REMAP, and ISSR uncovered reproducible polyploidization induced DNA rearrangements, measured as the lack of additive parental banding profiles in triticale. Nine of the ten IRAP, REMAP and ISSR primer combinations tested disclosed rearranged fragments. Only ISSR with primer (CA)9G produced a completely additive banding profile. Taken together, IRAP and REMAP with seven primer combinations and ISSR with three di-nucleotide sequences resulted in a total of 83 bands in the polyploid genotype, and 42 DNA sequence rearrangement events (51%). Of these, 36 corresponded to the loss of parental bands and 6 to the emergence of novel ones (Table 4). Interestingly, the intensities of monomorphic bands did not significantly differ between wheat and rye, suggesting that either the number of amplified *loci* or tandem repeats per *locus* are conserved between the two species. Using those markers we identified a higher proportion of rearrangements in triticale than the one observed in previous studies which is moreover exclusively associated with sequence modifications. Previous studies of polyploids using RFLP [9], [17] and AFLP [17] identified both genetic and epigenetic genome restructuring events, since cytosine methylation sensitive as well as non-sensitive restriction enzymes were used.

To our knowledge, IRAP and REMAP [25] and ISSR [26] have only been used once for polyploid analysis, when it was shown that parental genomes were highly conserved in the natural allopolyploid *Spartina anglica*. However, these molecular marker techniques designed by Kalendar and co-workers [27] to characterize cultivars and varieties in several species [27]–[29], not only provide an original approach to detect genetic rearrangements induced by polyploidization, but also contribute to a greater resolution of the specific sequences affected by polyploidy evolutionary process. The control experiment we designed with the wheat+rye test tube indeed reinforces that this technique is accurate in evaluating genomic restructuring events and detecting rearranged bands induced by polyplidization. Three rearranged fragments, representing the loss of a wheat parental band, loss of a rye parental band, and the appearance of a novel band in triticale were excised, purified and cloned for sequence analysis. The wheat-specific band (Mob-11-1200W) absent in triticale and obtained with IRAP showed significant homologies with sequences of *T. monococcum* (chromosome 7), *T. aestivum* cv Renan, *copia*-like retrotransposons previously identified in *Triticum*, namely *Claudia*, *Barbara*, *T. monococcum* 7Am (unnamed retrotransposon) and *Ty1-copia* from *O. sativa*. Alignment data further revealed this clone to be fragmented by the insertion of three different *copia*-like retrotransposons, and showed a 13 nucleotide polypurine-tract (PPT), which serves as a putative priming site for plus strand DNA synthesis [30]. A four nucleotide inverted repeat (TTGT) different to that of *Ty1*-*copia* and *Barbara* retrotransposons (TTGA) was identified immediately after the PPT region, indicating the presence of two classes of *copia* elements [31]. This clone could therefore represent a partial sequence of a retrotransposon with similarity to other elements, suggesting the occurrence of recombination events between partial sequences of single or multiple retroelements and/or families of retrotransposons. The rearranged rye fragment (Mob-111-1000R) absent in triticale and obtained by REMAP revealed homology with hydroxyproline-rich glycoproteins (HRGP) from *A. thaliana* and *O. sativa*. HRGP belong to a major family of inducible defence response proteins involved in the natural resistance of plants to injury, disease and various stress conditions [32], [33]. Perhaps the absence of this sequence in triticale is somehow related to its lower level of hardiness when compared to rye. The third sequence, isolated by REMAP, representing a novel band in triticale was found to be mostly microsatellite related, suggesting that polyploidization affected the number of these types of repetitive sequences.

The sequencing data provided important insights into which genomic sequences are involved in polyploidization induced modifications and cereal speciation. Retrotransposon-related genome rearrangements disclosed in this work were not restricted to non-coding regions, supporting the model proposed by Vitte and Panaud [19], where the organization of large plant genomes includes extensive heterochromatin blocks mainly composed of retrotransposons interspersed with gene-rich regions. During polyploidization, there may be a directed and controlled loss of coding sequences, a process believed to be necessary to overcome gene redundancy and achieve genetic diploidization. Accordingly, Southern blotting with probes to different genes indicated gene loss in synthetic allopolyploids of *Triticum* and *Aegilops* [7].Our banding profiles and sequencing results with LTR- and/or microsatellite-associated sequences suggest a direct role of retrotransposons in gene loss events. Although transcriptional activation of retrotransposons has been previously reported in wheat [7], [22] and *Arabidopsis* [13], [21], transposition of these elements has never been shown in newly synthesized polyploids.

The vast majority of rearrangements uncovered in the polyploid genotype corresponded to the loss of parental bands (86%, 36 out of 42, Table 4). This loss seems to have originated from sequence modification/elimination rather than a mutation in PCR primer binding sequences, since extensive sequencing of the LTR termini has shown that mutations are rare in these regions [34]. Moreover, studies on C values of various polyploids have indicated that genome downsizing might be a widespread phenomenon following polyploidy formation [35], and recently a reduction in DNA content was observed in six newly synthesized wheat allopolyploids [10]. Our results suggest that the decrease of DNA content previously reported in the course of triticale breeding [36] can be attributed to significant loss of retrotransposon and/or microsatellite flanking sequences. Our data showing that the majority of the missing bands in triticale corresponded to rye-specific bands (30 out of 36 lost bands), confirms previous data about the preferential occurrence of restructuring events in this parental genome [17]. This brings to mind the observed deletion of rye telomeric heterochromatin regions [37] responsible for the meiotic pairing failure and cytological instability of triticale [38]. Also reminiscent, albeit on another scale, is the preferential loss of 2R rye chromosome (the rye chromosome with greater DNA content) which usually results in more stable triticale plants with better agronomic characteristics [16].

Chromosome mapping with FISH on rye meristematic cells showed. REMAP products to be dispersed throughout the rye genome, but completely absent from centromeric domains and nucleolar organizing regions. Dot-like FISH signals clearly demonstrated that the labeled sequences were tandemly arranged and clustered, with marked accumulation in condensed sub-telomeric domains, suggesting their putative role in the establishment of terminal heterochromatin. The preferential disposition of FISH signals on telomeric domains is further enhanced by the Rabl organization of rye interphase nuclei, where major heterochromatic telomeric domains localize to one hemisphere. This disposition is similar to that observed for conserved domains of the *Ty1-copia*, *Ty3-gypsy* and *LINE* groups of retroelements on *Ae. speltoides*, and less obvious in *H. spontaneum*, probably due to the absence of telomeric heterochromatic domains in this species [39].

Differences in genome size, genetic redundancy and chromatin organization patterns between wheat and rye certainly resulted in genomic conflicts in the newly formed polyploid. In the triticale nucleus, rye has a greater haploid DNA content when compared to that of each genome complement from the hexaploid wheat [40]. Most likely, the preferential elimination of LTR and microsatellite-related sequences from rye played a role in homogenizing parental genomic DNA content. As previously mentioned, cytogenetic mapping of triticale showed dense heterochromatic domains in rye, with a high density of heterochromatin in sub-telomeric regions, that is not characteristic in wheat [41]. On the other hand, structural alterations in chromosome condensation often activate mechanisms such as DNA recombination and/or damage repair, leading to sequence excision/modification, as recently described in Drosophila mutant lines for genes coding chromatin remodeling enzymes [42]. Our molecular and cytogenetic results not only show the power of REMAP, IRAP and ISSR in uncovering polyploidization induced genetic restructuring events, but also allow for interesting interpretations regarding the genomic processes involved. It is tempting to speculate for instance that Retrotransposons -rich rye heterochromatic domains when present in triticale loose their capacity to remain condensed, allowing for the occurrence of changes in retrotransposon and microsattelite flanking sequences, essential for polyploid stability. The involvement of chromatin organization brings us back to the role of epigenetics on genetic adaptation and speciation. Future work will undoubtedly shed light into the complex interactions that occur between genetic and epigenetic events, and their role in molecular evolution.

## Materials and Methods

### Plant material and DNA isolation

The following plant material was used: synthetic octaploid primary triticale, (*Triticum aestivum* ‘Chinese Spring’×*Secale cereale* ‘Imperial’; 2n = 8x, AABBDDRR) at least 35 generations old, and their exact progenitors, hexaploid wheat *T. aestivum* ‘Chinese Spring’ (2n = 6x, AABBDD), and diploid rye *S. cereale* ‘Imperial’ (2n = 2x, RR). Although rye is naturally a highly outbreeding species and highly polymorphic genotype, the rye cultivar ‘Imperial’ used is highly inbreed and only inbred seeds has been maintained since 1944. Thus, parental species are very homozygous as a result of many years of selfing. Seeds stocks from all the genotypes were obtained from the USDA–Sears collection, Columbia, Mo. The ‘Chinese Spring’ wheat and ‘Imperial’ rye parents used in the present study were taken from the original seed envelopes used by E.R. Sears when he created the Chinese Spring/Imperial triticale. All seed stocks were germinated and grown in controlled conditions at a 16 hours light (20°C)/8 hours dark (20°C) cycle. Genomic DNA was isolated from fresh young leaves of eight week old plants using modified cetyltrimethylammonium bromide (CTAB) method [43]. For fluorescent *in situ* hybridization (FISH), three rye plants were analyzed separately. Root tips were collected from one week old plants, washed, fixed in ethanol/acetic acid (3:1 vol/vol) for 24h at room temperature, and stored at −20°C until use.

### IRAP and REMAP procedures

IRAP and REMAP PCR were performed in a 20 µl reaction mixture as previously detailed [27]. Primers for the LTR regions of four barley (*Hordeum vulgare* L.) retrotransposons [25] and three anchored microsatellite primers were utilized. A total of seven primer combinations were tested (Table 1). To assess if the rearranged bands detected in triticale are faithfully a result of polyploidization and did not result from competition between priming sites of one parent or the other, a mix of the parental wheat and rye DNA was used in the wheat+rye test tube.

### ISSR procedure

To determine the specific contribution of SSRs to the observed sequence rearrangements, ISSR amplifications were performed on the same plant material used for IRAP and REMAP. All three di-nucleotide repeats that were used for REMAP and IRAP were tested, and SSR primers are shown in Table 1. The amplification conditions were the same as for IRAP and REMAP and for all PCR experiments at least three technical replicates were completed.

### Electrophoresis and data analysis

PCR products were run on 1% agarose gels for 2–3 h at 110 volts, detected by ethidium bromide staining, and photographed using BioRad GEL DOC 2000. IRAP, REMAP and ISSR data were analyzed, and DNA bands were identified using the following criteria:

1. [i] Monomorphic bands: common to both triticale progenitors (wheat and rye);
2. [ii] Polymorphic bands: observed in only one triticale progenitor (wheat and rye);
3. [iii] Polyploid expected bands: expected from an additive profile of the characteristic wheat and rye parental bands;
4. [iv] Polyploid conserved bands: parental bands detected in the polyploid;
5. [v] Polyploid rearranged bands: parental (wheat and rye) bands absent in the polyploid as well as novel bands observed exclusively in the polyploid.

Once rearranged sequences were identified and verified for their reproducibility, several bands were gel-isolated, purified, cloned, and finally sequenced following the procedures described in Rocheta *et al.* [44]. The sequences obtained were used for BLAST on NCBI, TIGR, and PlantSat (repetitive plant sequences) databases as described in the results section. Cluster analysis were performed utilizing the Neighbor-Joining method [24] on the basis of a distance matrix calculated with the Bionumerics software (version 3.5).

### FISH

REMAP PCR reaction with primers CO699/(CT)9G was used as probe for *in situ* hybridization. The reaction conditions and amplification program were similar to those of normal REMAP, except that 1 µl (1 ηmol/µl) of digoxigenin-dUTP or biotin-dUTP (Roche, Gipf-Oberfrick, Switzerland) was added to the reaction mixture in order to label all PCR products. Ribosomal specific probe pTa71 [45] was used as control, labeled by nick translation using biotin-dUTP or digoxigenin-dUTP. Root tips were prepared as previously described for cytological analysis [46], with the following modifications. Fixed root tips were digested with pectinase/cellulase in 1×EB for 2h15min at 37°C, and squashes were performed in 60% glacial acetic acid. Nuclei and chromosomes were counterstained with 4′,6-diamidino-2-phenylindole hydrochloride (DAPI) in Citifluor antifade mounting medium (AF1; Agar Scientific). Samples were examined using a Zeiss Axioskop 2 epifluorescence microscope and images were obtained using a Zeiss AxioCam digital camera. Digital images were processed using PHOTOSHOP (Adobe Systems).

## Supporting Information

Figure S1 IRAP banding profiles - individual plants of each genotype. IRAP banding profiles obtained with primer Nikita from three individuals of each genotype: (W) Wheat, (R) Rye, and (T) Triticale.
(0.40 MB TIF)

Figure S2 IRAP banding profiles - wheat, rye, triticale and wheat+rye test tube. IRAP banding profile obtained with primer Nikita of wheat (W), rye (R), triticale (T), and wheat+rye test tube (W+R) showing triticale rearranged bands. Arrows indicate two rearranged band of rye genome origin absent in triticale but present in the wheat+rye test tube.
(0.16 MB TIF)
