# Supplementary figures and images for: Polyploidization as a Retraction Force in Plant Genome Evolution: Sequence Rearrangements in Triticale
Source: PLoS One. 2008 Jan 2;3(1):e1402. doi: 10.1371/journal.pone.0001402 (PMC2151762; doi:10.1371/journal.pone.0001402)

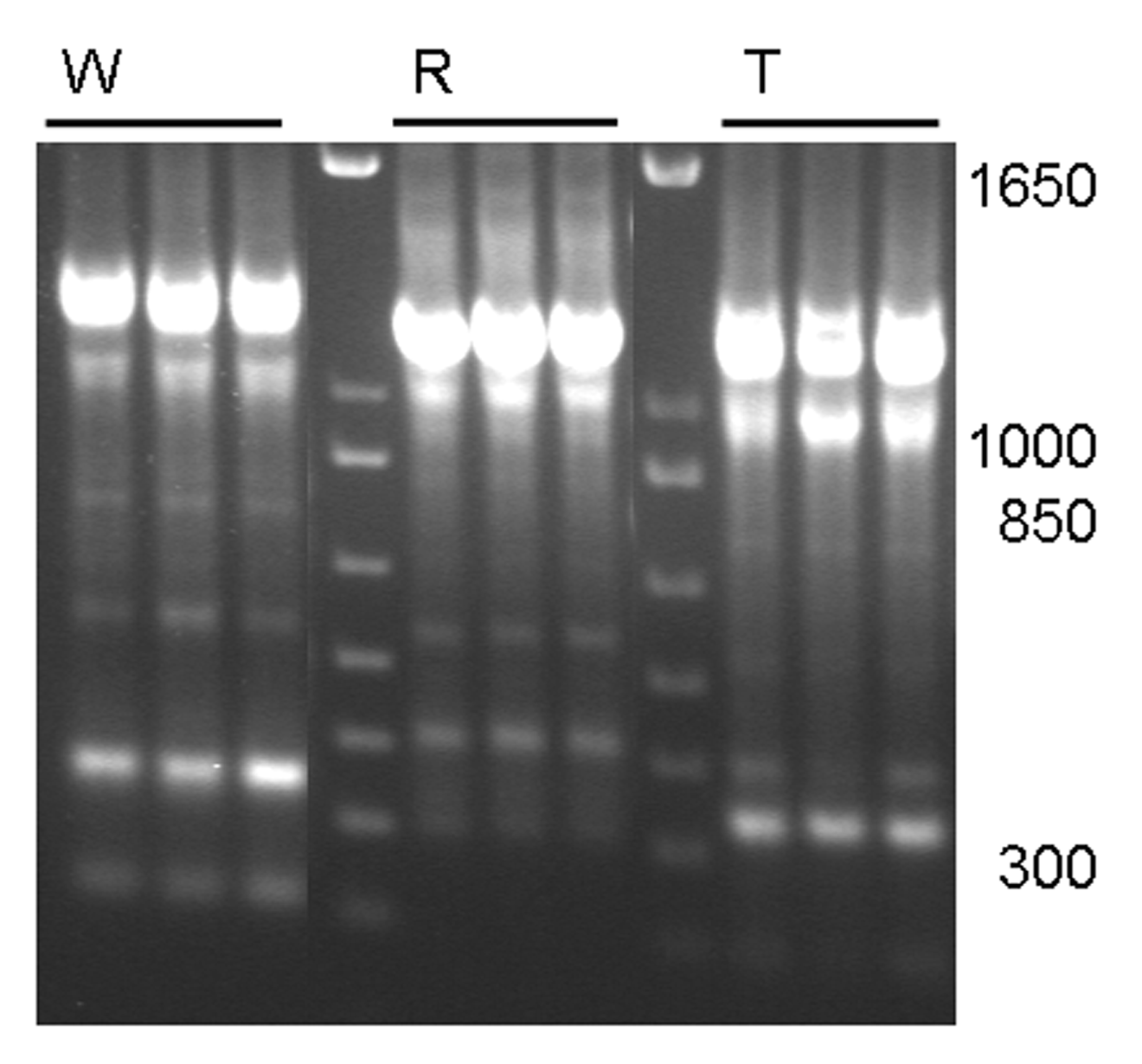

Supplement: Figure S1 — IRAP banding profiles - individual plants of each genotype. IRAP banding profiles obtained with primer Nikita from three individuals of each genotype: (W) Wheat, (R) Rye, and (T) Triticale. (0.40 MB TIF) [file pone.0001402.s001.tif]

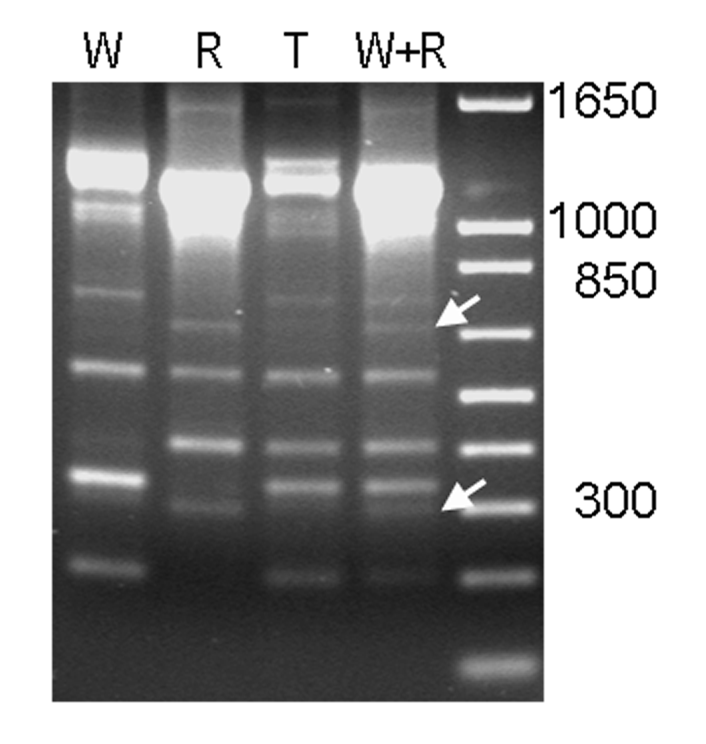

Supplement: Figure S2 — IRAP banding profiles - wheat, rye, triticale and wheat+rye test tube. IRAP banding profile obtained with primer Nikita of wheat (W), rye (R), triticale (T), and wheat+rye test tube (W+R) showing triticale rearranged bands. Arrows indicate two rearranged band of rye genome origin absent in triticale but present in the wheat+rye test tube. (0.16 MB TIF) [file pone.0001402.s002.tif]
